# The *Harvard Review of Psychiatry* Marks Its Twentieth Year

**DOI:** 10.3109/10673229.2012.652876

**Published:** 2012-02-15

**Authors:** Shelly F Greenfield

With the publication of this issue, the *Harvard Review of Psychiatry* celebrates the beginning of its twentieth year. We inaugurate this twentieth volume with a special issue devoted to global mental health that presents a perspective on the need to build capacity for diagnosis, treatment, research, and services delivery to resource-poor and culturally diverse regions across the world. As we initiate *HRP*'s twentieth year of publication, it seems appropriate for the *Review* to embrace the challenge of this critical issue of mental health in the delivery of twenty-first century health care.

Throughout its publishing history, *HRP* has sought to represent the depth and scope of psychiatry by presenting synthetic and thoughtful reviews and perspectives, as well as clinical challenges and columns, that highlight advances in our field from the basic sciences to clinical treatment to organization of services and education. The *Review*'s mission at the outset was to establish “a peer-reviewed publication that would provide succinct, readily understood, and authoritative reviews of current approaches to psychiatric diagnosis and treatment,” as well as “up-to-date reviews on topics … ranging from advances in human genetics to the application of new psychotherapeutic techniques.”^1^ As psychiatry experiences a continued exponential growth in new knowledge from molecular biology, genetics, neuroscience, clinical outcomes, epidemiology, and mental health services research, among many other fields, the *Review*'s role in providing current and accessible reviews of these advances to a broad audience of psychiatrists, trainees, and other mental health practitioners is arguably even more critical than when the *Review* published its first issue in January 1993.

*HRP* was among the first psychiatry journals to publish clinical case discussions in the form of its Clinical Challenges section—discussions that have consistently garnered attention from a wide array of psychiatrists and trainees. In recognition of issues regarding patient privacy, the *Review* was an early adopter of informed consent for patients whose clinical cases would be published, albeit in a format that disguised personally identifiable information. These clinical cases have presented the complexity of patients who are cared for throughout the mental health and general health care system in the United States and internationally, along with diverse and occasionally conflicting commentaries by clinical experts.

Beginning in 2001, *HRP* has published several special, themed issues each year. These issues have brought together a series of invited manuscripts focusing on new advances within the bounds of traditional psychiatric practice, as well as emerging issues in research and clinical care. The *Review* has offered special issues on wide-ranging topics, including contemporary issues in psychotherapy (2007), the neurobiology and treatment of substance use disorders (2004), mood stabilization from basic science to clinical practice (2003), emerging clinical challenges in child psychiatry (2008), updates in medical psychiatry (2009), opportunities and future challenges in forensic psychiatry (2010), late-life schizophrenia (2001), and updates on the pathophysiology and treatment of schizophrenia (2007). *The Review*'s special issues have also examined emerging issues in our field, including biomarkers and personalized psychiatry (2011), psychiatry and cyberspace (2010), women's mental health (2009), ethics and values in contemporary psychiatry (2008), the interface of genetics and clinical psychiatry (2006), advances in mental health services research (2006), and psychiatric responses to community trauma (2003).

Utilizing the format of briefer columns, *HRP* has presented articles in psychiatric education, the history of psychiatry both internationally and in the United States, psychiatric ethics, diagnostic nosology, neuroscience, cross-cultural psychiatry, women's issues, psychopharmacology, and substance use disorders, among many other areas. The *Review* has explored areas that are often understudied, although commonly encountered in clinical practice, by publishing articles on diverse topics such as the clinical care of siblings of patients with pervasive development disorders (2007), and the meaning and clinical relevance to parents of their child's attention-deficit/hyperactivity disorder diagnosis (2005). Putting our current approach to clinical observation and diagnosis into perspective, the *Review* published the first English translation and twenty-first century commentary on an 1882 German manuscript on “cyclic insanity”—an early grappling with clinical symptoms and presentation of bipolar mood disorder (2003).

As the *Harvard Review of Psychiatry* enters its twentieth year, its articles are increasingly cited in the psychiatry literature, with a recent journal impact factor of 2.2. The *Review* is widely read in both the United States and the rest of the world, and it continues to receive submissions of manuscripts from around the world. As we mark the twentieth year of the *Review*'s publication, we will continue its founding mission of encompassing the scope of our field and bringing accessible, scholarly reviews of the critical issues and advances in our field to a broad audience of psychiatrists and other mental health practitioners and trainees.
